# Efficient and Scalable Precision Genome Editing in *Staphylococcus aureus* through Conditional Recombineering and CRISPR/Cas9-Mediated Counterselection

**DOI:** 10.1128/mBio.00067-18

**Published:** 2018-02-20

**Authors:** Kelsi Penewit, Elizabeth A. Holmes, Kathyrn McLean, Mingxin Ren, Adam Waalkes, Stephen J. Salipante

**Affiliations:** a Department of Laboratory Medicine, University of Washington, Seattle, Washington, USA; b Department of Bioengineering, University of Washington, Seattle, Washington, USA; University of Delaware

**Keywords:** CRISPR, Cas9, *Staphylococcus aureus*, genetic engineering, genome editing, *mutS*, recombineering

## Abstract

*Staphylococcus aureus* is an important human pathogen, but studies of the organism have suffered from the lack of a robust tool set for its genetic and genomic manipulation. Here we report the development of a system for the facile and high-throughput genomic engineering of *S. aureus* using single-stranded DNA (ssDNA) oligonucleotide recombineering coupled with clustered regularly interspaced short palindromic repeat (CRISPR)/Cas9-mediated counterselection. We identify recombinase *EF2132*, derived from *Enterococcus faecalis*, as being capable of integrating single-stranded DNA oligonucleotides into the *S. aureus* genome. We found that *EF2132* can readily mediate recombineering across multiple characterized strains (3 of 3 tested) and primary clinical isolates (6 of 6 tested), typically yielding thousands of recombinants per transformation. Surprisingly, we also found that some *S. aureus* strains are naturally recombinogenic at measurable frequencies when oligonucleotides are introduced by electroporation, even without exogenous recombinase expression. We construct a temperature-sensitive, two-vector system which enables conditional recombineering and CRISPR/Cas9-mediated counterselection in *S. aureus* without permanently introducing exogenous genetic material or unintended genetic lesions. We demonstrate the ability of this system to efficiently and precisely engineer point mutations and large single-gene deletions in the *S. aureus* genome and to yield highly enriched populations of engineered recombinants even in the absence of an externally selectable phenotype. By virtue of utilizing inexpensive, commercially synthesized synthetic DNA oligonucleotides as substrates for recombineering and counterselection, this system provides a scalable, versatile, precise, inexpensive, and generally useful tool for producing isogenic strains in *S. aureus* which will enable the high-throughput functional assessment of genome variation and gene function across multiple strain backgrounds.

## IMPORTANCE

Engineering genetic changes in bacteria is critical to understanding the function of particular genes or mutations but is currently a laborious and technically challenging process to perform for the important human pathogen *Staphylococcus aureus*. In an effort to develop methods which are rapid, easy, scalable, versatile, and inexpensive, here we describe a system for incorporating synthetic, mutagenic DNA molecules into the *S. aureus* genome and for eliminating cells that lack the engineered mutation. This method allows efficient, precise, and high-throughput genetic engineering of *S. aureus* strains and will facilitate studies seeking to address a variety of issues about the function of particular genes and specific mutations.

## INTRODUCTION

*Staphylococcus aureus* is a common and highly successful opportunistic pathogen that is responsible for a high burden of patient morbidity and mortality nationwide and worldwide (1). *S. aureus* underlies diverse clinical diseases, ranging from relatively benign to life-threatening diseases, involving many different organ systems (2–4). Moreover, *S. aureus* infections, especially noncutaneous infections, are often chronic or relapsing (5–8) and are difficult to eradicate permanently. Despite its medical importance, knowledge of key factors which promote virulence, chronicity, and pathogenicity in *S. aureus* remains incomplete (9).

Genes contributing to clinically relevant phenotypes in *S. aureus* are believed to be numerous (10–12) and are frequently regulated in large, multicomponent networks (13, 14). Yet, *S. aureus* is difficult to manipulate genetically, making it challenging to experimentally test the effects of specific genes or mutations through the construction of isogenic strains. A major advance in the field was heralded by the development of methods to bypass common *S. aureus* restriction systems, which serve as strong barriers to the introduction of exogenous DNA. The construction of a transgenic cytosine methylase-negative *Escherichia coli* strain first enabled escape of the *S. aureus* type IV restriction system (15), and the strain’s subsequent modification to actively mimic methylation profiles of major *S. aureus* clonal complexes (CCs) further improved efficiencies by avoiding the type III restriction systems (16). Whereas previously only strains carrying spontaneous or induced mutations in their restriction systems could be manipulated, these technologies have now enabled high-efficiency transformation of engineered plasmids into most laboratory and clinical *S. aureus* strains.

Most established methods for modifying *S. aureus* genomes rely on rare homologous recombination events with large donor fragments encoding the desired change, with antibiotic-mediated selection for successful allelic exchange (17–19). Although useful, these techniques are relatively inefficient and introduce exogenous genetic material into the host genome along with the targeted mutation (18, 20). Modified approaches utilizing lethal, counterselectable markers, including antisense-*secY* expression (21), toxic metabolites (19), homing endonucleases (19), and, most recently, clustered regularly interspaced short palindromic repeat (CRISPR)/Cas9 (22, 23), have increased enrichment for genetically modified strains to various degrees or have eliminated the need for the introduction of chromosomally integrated markers. Nevertheless, all available genome editing strategies for *S. aureus* remain laborious and involve the individual cloning of each ~1-kb-to-2-kb homologous repair template, with accompanying protocol optimization (17, 18, 20–22, 24); this strategy is especially difficult to implement for engineering gene deletions, which must be manufactured using splicing by overhang extension (SOE) PCR (15, 23).

To address the methodological need for a facile, versatile, and scalable system for *S. aureus* precision genomic engineering, here we have developed conditional systems for recombineering (25) and CRISPR/Cas9-mediated counterselection (26) in that organism. First pioneered in *E. coli* (27), this powerful strategy has subsequently been adapted to other species, including Gram-positive organisms (28–32), although the range of bacteria for which such tools exist remains limited (32). Recombineering, which incorporates mutagenic oligonucleotides into a host genome through the action of bacteriophage-derived single-stranded DNA (ssDNA) recombinases, allows point mutations, variable-length deletions, and small insertions to be precisely engineered using short, commercially synthesized oligonucleotides (25, 31, 33, 34). Following recombineering, CRISPR/Cas9-mediated endonuclease cleavage targeted to the wild-type allele provides counterselection for the engineered change, even in the absence of an externally selectable phenotype, by introducing lethal double-stranded DNA breaks into the genome of unedited cells (27). Because recombineering and counterselection vector construction can be performed using inexpensive, commercially manufactured ssDNA fragments, the system is inherently scalable and amenable to high-throughput applications.

## RESULTS

### Identification of a single-stranded oligonucleotide recombineering protein with activity in *S. aureus*.

**FIG 1:**
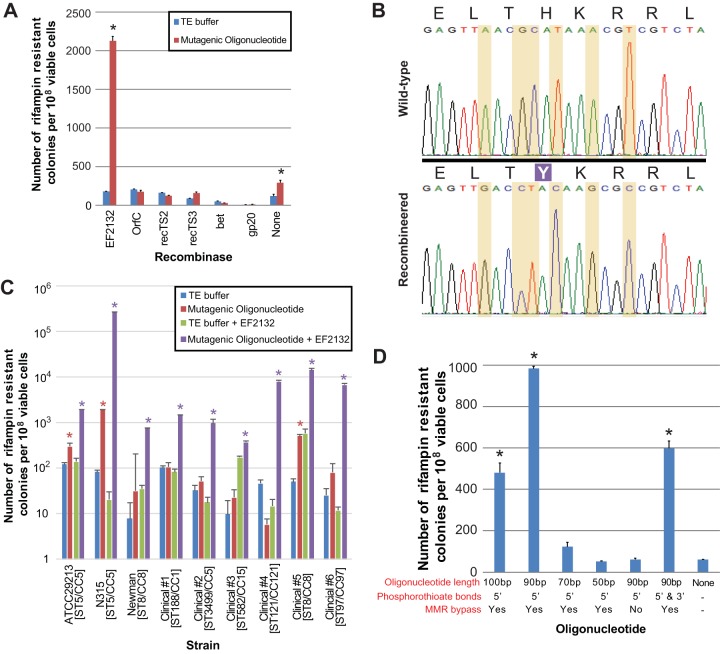
Recombineering in *S. aureus*. (A) Activity of different recombinases when expressed exogenously in *S. aureus* compared to paired, mock-transformation controls lacking mutagenic oligonucleotide. Recombineering utilized mutagenic oligonucleotides encoding rifampin resistance. Recombination frequencies significantly higher than those observed in paired controls (≤*P* = 0.002 [two-tailed *t* test]) are indicated by an asterisk. TE, Tris-EDTA. (B) Sequence confirmation of recombineered isolates. Residues 477 through 485 of *S. aureus rpoB* are shown, with the H481Y missense mutation indicated as a white letter in a purple field. Mutations introduced by the recombineering oligonucleotide are highlighted between wild-type and engineered strains. (C) Recombineering efficiencies across laboratory and clinical strains. Results are shown for wild-type strains and strains exogenously expressing recombinase EF2132 compared to paired mock-transformation controls. Recombination frequencies significantly higher than those observed in controls (≤*P* = 0.008 [two-tailed *t* test]) are indicated by a red asterisk for wild-type strains and a purple asterisk for strains expressing recombinase. Sequence type (ST) and clonal complex (CC) data are indicated in brackets next to each strain name. (D) Effect of various oligonucleotide lengths, mismatch repair-evading silent substitutions, and phosphorothioate modifications on recombination efficiency. Recombination frequencies significantly higher than those observed in the mock transformation control (≤*P* = 0.002 [two-tailed *t* test]) are indicated by an asterisk. Error bars in all panels indicate standard errors of the means.

We initially tested the activity of various known and predicted recombinases in *S. aureus* by evaluating their ability to mediate ssDNA recombineering of the *rpoB* H481Y mutation, which confers rifampin resistance (35). We evaluated six different recombinases exogenously expressed from cassette-based shuttle vector pCN50 (36): *bet*, the recombinase gene utilized in the *E. coli* λ Red recombineering system (25); *EF2132* and *orfC*, derived from *Enterococcus faecalis* and *Legionella pneumophilia*, respectively, both being genes that have been previously shown to have cross-species activity in *E. coli* (25); *gp20*, a recombinase originating from *S. aureus* with weak activity in *E. coli* (25); and two putative *S. aureus* recombinases (which we termed *recTS2* and *recTS3*) which we identified on the basis of protein homology to these known recombinases. All proteins were codon optimized for expression in *S. aureus*. Each expression construct was separately introduced into *S. aureus* type strain ATCC 29213 (Rosenbach [37]), electrocompetent cells were prepared, and mutagenic oligonucleotide was subsequently introduced by electroporation. Recombineering activity was evaluated by comparing the number of rifampin-resistant colonies produced using the mutagenic oligonucleotide to the number seen after mock electroporation in the absence of the oligonucleotide (Fig. 1A).

Only *EF2132* achieved recombineering frequencies significantly (*P* = 3.2 × 10^−5^ [two-tailed *t* test]) greater than the rate of spontaneous rifampin resistance occurring in mock-transformation and recombinase-negative controls, ~2 × 10^−5^ per cell (Fig. 1A). Confirmatory sequencing of 10 rifampin-resistant recombinants showed that each carried all six mutations encoded by the mutagenic oligonucleotide (two substitutions encoding H481Y plus four silent changes to escape reversion by mismatch repair [MMR] [38]) (Fig. 1B), indicating that the phenotype resulted from incorporation of the synthetic oligonucleotide into the genome rather than from spontaneous, resistance-conferring point mutations. Surprisingly, rifampin-resistant colonies were obtained at approximately twice the spontaneous background mutation rate (*P* = 0.002 [two-tailed *t* test]) when recombineering was performed in the absence of exogenous recombinase expression, suggesting that strain ATCC 29213 is naturally recombinogenic at low frequencies.

To assess the generality of *EF2132*-mediated recombineering function, we repeated the assay in laboratory strain Newman (39), previously characterized clinical isolate N315 (40), and six randomly selected and otherwise uncharacterized primary clinical *S. aureus* isolates which were obtained from unrelated patient specimens (arbitrarily numbered 1 to 6; Fig. 1C).

Strains ATCC 29213, Newman, and N315 were each transformable with recombineering plasmid passaged through a transgenic *E. coli* strain that modifies plasmid DNA to confer the adenine methylation profile of *S. aureus* CC8 (16), consistent with the compatible restriction groups of these isolates’ clonal complexes (CC5 and CC8; see Table S1 in the supplemental material). Recombineering in *S. aureus* laboratory strain Newman produced statistically significant numbers of recombinants only in the presence of exogenous recombinase expression and achieved a recombineering rate approximately half that seen for ATCC 29213 (7.32 × 10^−6^ recombinants per cell; *P* = 1.20 × 10^−4^ [two-tailed *t* test]). In contrast, strain N315 achieved recombineering efficiencies (2.5 × 10^−3^ recombinants per cell; *P* = 8.06 × 10^−6^ [two-tailed *t* test]) that were nearly 2 orders of magnitude greater than those seen with ATCC 29213 and which similarly yielded statistically significant numbers of recombinants in the absence of exogenous recombinase expression (1.02 × 10^−6^ per cell; *P* = 1.66 × 10^−8^ [two-tailed *t* test]).

10.1128/mBio.00067-18.2 TABLE S1  Summary of strains and plasmids used. Download TABLE S1, XLSX file, 0.02 MB. Copyright © 2018 Penewit et al. 2018 Penewit et al. This content is distributed under the terms of the Creative Commons Attribution 4.0 International license.

Five of the clinical strains (strains 1, 2, 3, 5, and 6) were transformable with recombineering vector after plasmid artificial modification to actively mimic CC8 adenine methylation (16); using whole-genome analysis, these strains were subsequently determined to belong to CC8 and restriction-compatible groups CC1, CC5, CC15, and CC97 (Table S1). The remaining isolate (strain 4) was transformable only with vector lacking cytosine methylation (15) and was later ascertained to be a member of CC121, explaining its incompatibility with CC8-modified plasmid. All six of the clinical strains carrying recombineering vector and transformed with mutagenic oligonucleotide generated rifampin-resistant colonies at statistically significantly higher frequencies than mock transformations lacking the recombineering oligonucleotide (*P* = 7.6 × 10^−3^ to 4.2 × 10^−5^ [two-tailed *t* test]), with efficiencies varying by 2 orders of magnitude (range, 3.66 × 10^−6^ to 1.4 × 10^−4^ recombinants per cell). Clinical strain 5 alone yielded statistically significant numbers of recombinants in the absence of exogenous recombinase expression (5.0 × 10^−6^ per cell; *P* = 1.6 × 10^−4^ [two-tailed *t* test]).

Collectively, these data both indicate that expression of *EF2123* is able to generally catalyze recombineering across different *S. aureus* strain backgrounds, regardless of clonal complex or sequence type, albeit at various efficiencies, and reinforce the idea that some isolates are naturally recombinogenic without the expression of exogenous recombinase.

The N315 genome includes an endogenous bacteriophage, ϕN315 (41), and the draft assembly of ATCC 29213 similarly contains evidence of a lysogenic phage sequence (42). We reasoned that the ability of some strains to natively recombineer with ssDNA oligonucleotides could reflect contributions from bacteriophage-carried genes present in their genomes. We therefore evaluated the recombineering capacity of an N315 background from which ϕN315 had been deleted (43). Compared to the parental strain, no reduction in recombineering frequency was observed. This result suggests that currently undefined host-encoded factors, rather than endogenous bacteriophage proteins, influence recombineering efficiencies in *S. aureus*.

### Optimization of recombineering oligonucleotides.

Various properties and modifications of oligonucleotides have been reported to influence recombineering efficiency in *E. coli*, including length (38), configuration of phosphorothioate bonds which inhibit exonuclease digestion (44), and inclusion of mismatches to escape reversion of changes engineered by the mismatch repair (MMR) system (38). To explore how each of these considerations influences recombineering in *S. aureus*, we evaluated oligonucleotides which differed in one or more of these features (Fig. 1D).

Optimal recombineering frequencies were obtained with oligonucleotides of 90 bp in length, significantly exceeding the length previously reported to be optimal for *E. coli* (38). Recombineering efficiency was reduced approximately 2-fold when using 100-bp oligonucleotides, 10-fold for templates of 70 bp, and was not significantly different from background rifampin resistance rates when using 50-bp oligonucleotides. Incorporating phosphorothioate bonds into the oligonucleotide 5′ end yielded the greatest number of recombinants in *S. aureus*, in comparison to inclusion of that modification on both ends, which is optimal for *E. coli* (44). Exclusion of additional, silent mutations intended to bypass MMR correction of engineered changes (38) resulted in recombineering efficiencies that were indistinguishable from background mutation levels, indicating that the MMR system represents a major barrier to genome engineering in *S. aureus*.

In summary, we found that 90-bp oligonucleotides carrying 5′ phosphorothioate bonds and modifications to escape the MMR system promote recombineering most efficiently in *S. aureus*.

### Developing a two-plasmid system for conditional recombineering and counterselection.

**FIG 2:**
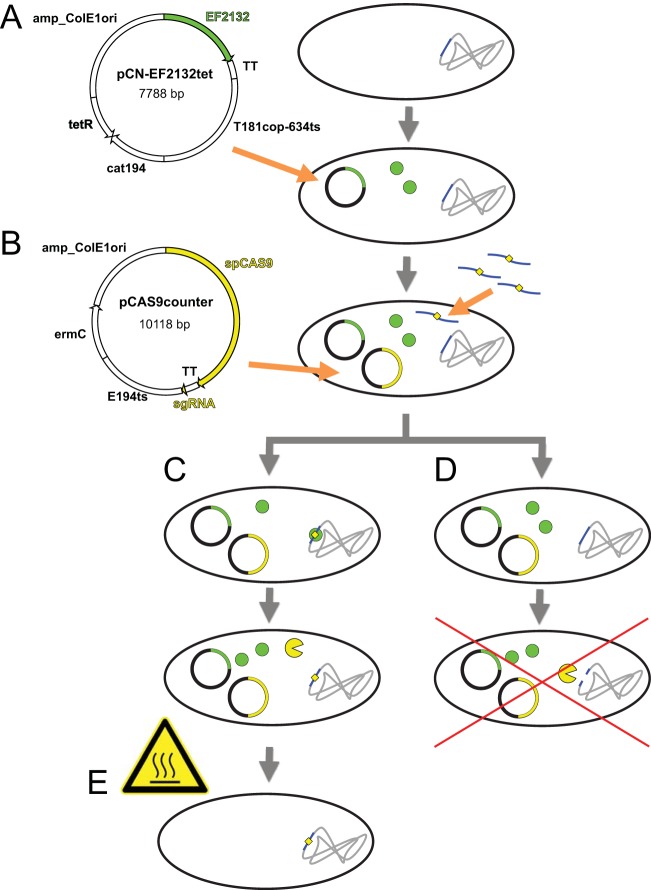
Overview of recombineering and Cas9-mediated counterselection for *S. aureus* genome engineering. (A) Temperature-sensitive recombineering vector pCN-EF2132tet, encoding recombinase (green circles), is transformed into the strain to be edited. Key elements of the vector are diagrammed and include recombinase EF2132 followed by a transcriptional terminator; high-copy-number temperature-sensitive *S. aureus* replicon T181cop-634ts; a chloramphenicol resistance gene for selection in *S. aureus*; and the *E. coli ColE1* origin of replication with an ampicillin resistance selectable marker for maintenance in *E. coli*. (B) Temperature-sensitive counterselection plasmid pCAS9counter is introduced at the time that recombineering is performed. Key elements of the vector are diagrammed and include a synthetic guide RNA (sgRNA) targeted to the genomic site being modified; Cas9 followed by a transcriptional terminator; low-copy-number temperature-sensitive *S. aureus* replicon E194ts (which is compatible with T181cop-634ts); an erythromycin resistance gene for selection in *S. aureus*; and the *E. coli ColE1* origin of replication with an ampicillin resistance selectable marker for maintenance in *E. coli*. (C and D) Recombineering is performed using a mutagenic oligonucleotide (blue curved lines) encoding the desired change (yellow diamond). Transformation with these elements leads to two possible outcomes: integration of the mutagenic oligonucleotide is successful and Cas9 (yellow Pac-Man symbol) is unable to cleave the genome (C), or integration of the mutagenic oligonucleotide does not occur and Cas9 introduces a double-stranded break into the host genome, killing unedited cells (D). (E) Brief growth of bacteria at elevated temperatures which are nonpermissive for the plasmid system results in its loss, leaving isogenic, edited cells.

To enable recovery of recombineered isolates without a selectable phenotype, we next developed a temperature-sensitive, two-vector system to carry out conditional recombineering and CRISPR/Cas9-mediated counterselection in *S. aureus* (Fig. 2). The pCN-EF2132tet recombineering vector expresses *EF2132* recombinase on a chloramphenicol selectable backbone. The pCas9counter counterselection vector confers erythromycin resistance and expresses a self-sufficient small guide RNA (sgRNA) (45) and Cas9. Targeting of Cas9 to a locus of interest is achieved by cloning a 20-bp DNA oligonucleotide (protospacer element) into the sgRNA backbone which matches the specified locus and is immediately upstream of a Cas9 recognition site (protospacer adjacent motif [PAM] 5′-NGG-3′). Both constructs are built on shuttle vectors and therefore can be propagated and genetically manipulated in *E. coli*. Genome engineering is accomplished by first transforming pCN-EF2132tet into a given *S. aureus* strain to make a recombineering-competent lineage and then subsequently introducing the recombineering oligonucleotide concurrently with the counterselection vector. Only cells undergoing successful recombineering are immune to lethal, double-stranded DNA breaks induced by the counterselection plasmid such that erythromycin resistance serves as a selectable proxy for the recombineered change. The growth temperature of engineered cells can be subsequently increased, resulting in loss of the vector system.

We tested the efficacy of our Cas9-mediated counterselection strategy in ATCC 29213, a type strain which exhibited representative recombineering efficiencies for *S. aureus* (Fig. 1C). Recombineering utilized an oligonucleotide encoding both *rpoB* H481Y and a noncoding mutation which eliminated a nearby PAM, with counterselection targeted to that ablated site. Given this design, successful recombineering with the oligonucleotide provides both rifampin resistance and immunity to Cas9-mediated counterselection, allowing the effects of recombineering and Cas9 activity to be separately investigated.

**FIG 3:**
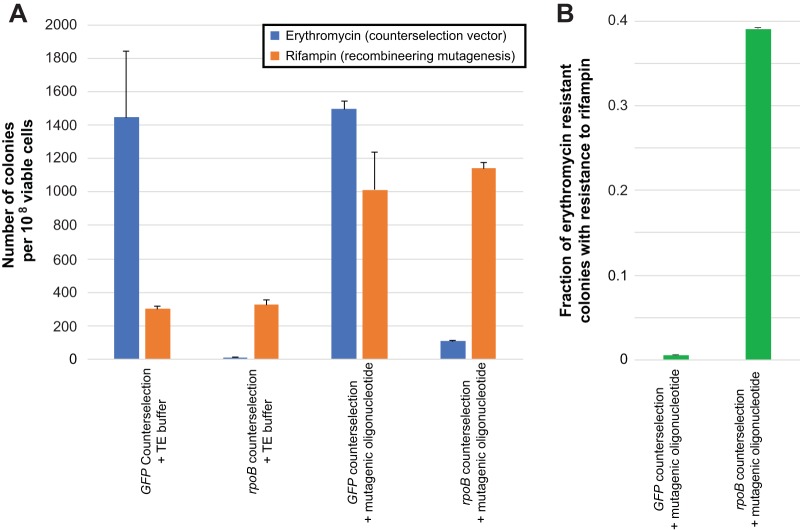
Recombineering with concurrent Cas9-mediated counterselection. (A) Frequency of colonies recovered using different combinations of counterselection vectors, rifampin resistance recombineering oligonucleotide, and selective media when recombineering oligonucleotides and counterselection vectors are introduced concurrently. (B) Fraction of colonies carrying counterselection vectors which display resistance to rifampin after recombineering with cotransformation of counterselection vectors. Error bars in both panels indicate standard errors of the means.

Consistent with our earlier experiments (Fig. 1C), limited numbers of rifampin-resistant colonies were obtained when *S. aureus* carrying the recombineering vector was transformed with counterselection plasmids targeted either to an irrelevant biological sequence (*GFP*) or the *rpoB* PAM site (Fig. 3A), indicating a low background frequency of spontaneous rifampin resistance. Transformation with the *rpoB* counterselection vector, however, yielded less than 1% of the erythromycin-resistant colonies obtained from the *GFP*-targeted control (*P* = 0.04 [two-tailed *t* test]), demonstrating that the vast majority of cells taking up active counterselection plasmid were killed by it. When recombineering was performed during cotransformation of the *GFP*-targeted counterselection vector, colony counts on rifampin-containing medium were also consistent with prior recombineering efficiencies, with slightly higher counts of erythromycin-resistant colonies suggesting higher efficiencies of plasmid transformation than recombineering. When recombineering was performed during cotransformation of the counterselection vector targeted to *rpoB*, the number of recombinants that were recovered on rifampin media were equivalent to those seen when the *GFP*-targeted construct was used. Although the numbers of erythromycin-resistant colonies were markedly reduced compared to the numbers seen with the *GFP*-targeted control (*P* = 3.0 × 10^−5^ [two-tailed *t* test]), those counts were significantly elevated compared to the viable colonies seen with transformation of the *rpoB* counterselection vector alone (*P* = 1.1 × 10^−4^ [two-tailed *t* test]), evidencing increased survival in the context of recombineering.

In contrast to the *GFP*-targeted control, replica plating of recombineered, *rpoB*-counterselected cells onto rifampin-containing media indicated that the population was significantly enriched (*P* = 3.2 × 10^−3^ [two-tailed *t* test]) for rifampin-resistant recombinants (Fig. 3B). However, only a fraction of erythromycin-resistant colonies were resistant to rifampin, and we sequenced counterselected isolates at the site of recombineering to further investigate. Of 12 colonies that were resistant to rifampin and erythromycin, 10 carried both the mutations conferring rifampin resistance and those ablating the PAM, consistent with the intended recombineering product, while the remaining two were wild type, suggesting separate escape mutations which inactivated the counterselection plasmid and conferred rifampin resistance. A total of 9 of 10 erythromycin-resistant colonies which were sensitive to rifampin were similarly wild type, while 1 harbored only the ablated PAM mutation. Collectively, these results indicate that >30% of all isolates surviving counterselection represented the intended recombinants and that recombinants can be dramatically enriched using counterselection alone.

To confirm temperature-sensitive loss of the plasmid system, we tested colonies containing both vectors for their retention of vector-conferred antibiotic resistance phenotypes after growth on nonselective media overnight under temperatures that were permissive (32°C) or nonpermissive (43°C) for plasmid replication. All 10 colonies grown at the permissive temperature retained resistance to both chloramphenicol and erythromycin, whereas all 10 of the colonies grown at the nonpermissive temperature were unable to grow in the presence of either antibiotic, indicating effective loss of both plasmids after brief passaging at elevated temperatures.

### Recombineering of a *mutS* gene deletion allows high-efficiency recombineering of single nucleotide substitutions.

To expand the utility of our genome editing system in *S. aureus*, and to demonstrate recombineering of a large deletion lacking a selectable phenotype, we sought to generate an MMR-deficient strain which would be capable of recombineering single base changes with high efficiency (46). We therefore engineered into ATCC 29213 a 2,484-bp (828-amino-acid), in-frame *mutS* deletion (residues 16 to 843) using a mutagenic oligonucleotide matching 45 bp of flanking sequence on either side of the change, with concurrent counterselection performed against a PAM within the deleted region.

**FIG 4:**
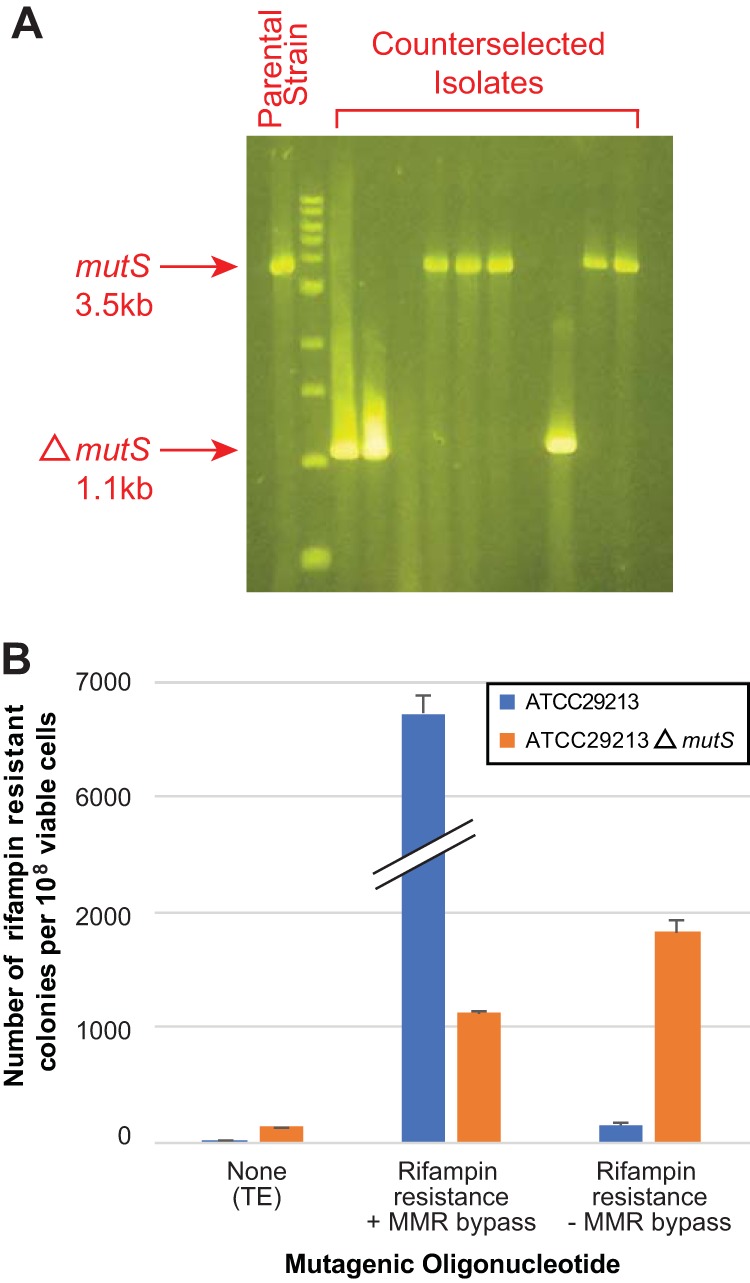
Recombineering of a 2.4-kb *mutS* deletion strain and effects of *mutS* deletion on subsequent recombineering. (A) PCR amplification of the *mutS* locus from representative colonies surviving counterselection. The wild-type allele corresponds to an amplicon 3.5 kb in size, whereas the engineered deletion results in a product at 1.1 kb. A 1-kb ladder is shown. (B) Recombineering efficiencies of the *mutS* deletion strain and *mutS*-intact parental strain compared to paired, mock-transformation controls lacking mutagenic oligonucleotide. Recombineering oligonucleotides encode rifampin resistance and either incorporate or lack silent mutations promoting bypass of MMR pathway repair. Error bars indicate standard errors of the means.

Viable colonies carrying the counterselection vector were obtained at a rate of 13 ± 9.1 recombinants (average ± standard deviation) per 10^8^ cells, roughly one-quarter the frequency obtained when recombineering base substitution mutations. Successful editing events were evaluated by fragment length analysis PCR (Fig. 4A). Of the 10 colonies screened, three (~30%) carried a size shift consistent with the deletion. Half (50%) were wild type and thus likely resulted from spontaneous mutation of the Cas9 target site or the counterselection plasmid itself. The remaining two colonies failed to amplify with primers exterior to the *mutS* gene, suggesting a deletion of the locus beyond the intended boundaries.

As a final step, we evaluated the ability of the *mutS*-deficient strain to recombineer mutagenic oligonucleotides either containing or lacking silent mutations designed to evade MMR repair when carrying the recombineering vector (Fig. 4B). Surprisingly, the *mutS* deletion mutant was able to recombineer an oligonucleotide encoding *rpoB* H481Y and MMR-bypassing synonymous mutations with only one-sixth the efficiency of the parental strain (*P* = 8.7 × 10^−6^ [two-tailed *t* test]). However, while the parental strain could incorporate oligonucleotides lacking MMR bypass mutations at rates of ~150 per 10^8^ cells, only modestly (*P* = 0.014 [two-tailed *t* test]) higher than the background mutation frequency, the *mutS* deletion strain produced recombinants with significantly higher rates (*P* = 1.4 × 10^−5^ [two-tailed *t* test]) approaching 2,000 per 10^8^ viable cells. These rates were also markedly greater (*P* = 4.6 × 10^−6^ [two-tailed *t* test]) than those seen for the *mutS* deletion strain when recombineering was performed using oligonucleotides containing MMR-bypassing synonymous mutations, likely reflecting the decreased number of mismatches between the mutagenic oligonucleotide and the genome. As expected from the role of *mutS* in repairing errors arising during DNA replication, we observed more than a 7-fold increase in the rate of spontaneous rifampin-resistant isolates for the *mutS* deletion strain compared to the wild type (*P* = 1.5 × 10^−4^ [two-tailed *t* test]) when mock transformation was performed, consistent with a mutator phenotype (47). We conclude that the *mutS*-deficient background allows robust recombineering of single base mismatches, at the expense of an increased background mutation rate.

### Efficiency of gene deletion by recombineering.

To explore the editing efficiencies of additional genes across the *S. aureus* genome, we recombineered in-frame deletions for seven additional factors (*aroE*, *baeS*, *cyoD*, *nupC*, *purR*, *ypfH*, and *yhcF*) in ATCC 29213, performing counterselection and screening as before. There was substantial variability in the performance of the individual editing events (see Fig. S1 in the supplemental material), in terms of both the fraction of counterselected colonies carrying the desired edit (range, 0.1 to 0.875) and the rate of successful editing events per viable cell (3.2 × 10^−9^ to 2.3 × 10^−7^). Consistent with recombineering studies in *E. coli* (48), we observed a negative correlation between the size of the engineered deletion and the efficiency with which it could be generated. However, even deletions of similar sizes could display marked differences in editing efficiency, suggesting that other factors, such as local genomic architecture and recombineering oligonucleotide properties, have agency (48).

10.1128/mBio.00067-18.1 FIG S1  Efficiency of constructing in-frame deletion mutants. Success rates for constructing the desired in-frame deletions are indicated as a function of deletion size. Results are expressed as the fraction of counterselected colonies carrying the desired edit (left *y* axis, blue data series) and the rate of successful editing events per viable cell (right *y* axis, orange data series). Gene names are indicated above relevant data points. Download FIG S1, PDF file, 0.99 MB. Copyright © 2018 Penewit et al. 2018 Penewit et al. This content is distributed under the terms of the Creative Commons Attribution 4.0 International license.

## DISCUSSION

Here we describe a simple, precise, efficient, scalable, rapid, and inexpensive method for engineering directed genomic changes in *S. aureus*, related to systems which have been developed for other organisms (27–32). We have demonstrated its utility in generating both point mutations (Fig. 1) and large gene deletions (Fig. 4). Our method (Fig. 2) entails successively transforming *S. aureus* with a recombineering vector to make a conditionally recombinogenic strain and then performing recombineering in the presence of a locus-specific CRISPR/Cas9-mediated counterselection vector, which results in the elimination of unedited cells. By virtue of being encoded on temperature-sensitive elements, this system allows genome editing to be performed without the permanent carriage of selectable markers or genome editing machinery, and it can consequently be used to produce truly isogenic *S. aureus* strains. In contrast to existing genome modification systems in *S. aureus* (17, 18, 20–22), a major advantage of our approach is that it both generates and selects for desired genetic changes using inexpensive, commercially synthesized synthetic single-stranded DNA oligonucleotides, which can be manufactured in bulk. As such, this technology is amenable to scaling and therefore provides opportunities for high-throughput genome engineering in *S. aureus*. Moreover, recombineering has also proven highly efficient, typically yielding hundreds to thousands of recombinants per transformation (Fig. 1C) or 10 to 20 recombinants during cotransformation of counterselection constructs (Fig. 3). Recombineering can be generally performed in *S. aureus* lineages which can be made electrocompetent, and have we have shown functionality in both laboratory and clinical strains (Fig. 1C). We have found that recombineering efficiencies can vary markedly from strain to strain, based on contributions of host factors which have not yet been identified. Counterselection results in populations which are highly enriched for successful recombinants (Fig. 3B), which accounted for ~30% of the surviving population when point mutations or a large gene deletion were engineered.

The workflow of our approach is swift, even when it is performed in a *S. aureus* strain for the first time. Counterselection vectors are readily constructed in advance from synthetic oligonucleotides and are cloned directly into transgenic *E. coli* strains that modify plasmids to escape *S. aureus* restriction systems (15) (day 1). Successful clones are rapidly identified by fragment length analysis PCR (day 2), and plasmid is harvested (day 3). Preparing electrocompetent cells for a given *S. aureus* strain and transformation with the recombineering vector are performed only once (day 1), after which transgenic electrocompetent cells are prepared (day 2). This single batch of electrocompetent cells can enable transformation of many different mutagenic oligonucleotides and counterselection vectors (day 2), with screening of recombinants possible the following day (day 3).

Because expression of CRISPR/Cas9 targeted to the genome is lethal in wild-type strains, effective regulation of counterselection constructs is critical (34). We initially attempted to achieve regulation of counterselection using an inducible system (34) but were unable to achieve complete repression of Cas9 activity even when using a mutagenized promoter that minimized leakiness (49). Regardless, the high efficiencies achievable by our system allowed simultaneous introduction of the recombineering oligonucleotide and the counterselection construct to achieve concurrent genome editing and counterselection. This configuration also eliminates the need to prepare electrocompetent lines after introduction of each counterselection vector, facilitating higher throughput.

Our work has revealed several interesting features of *S. aureus* biology as relevant to recombineering. First, we were unable to identify a recombinase originating from *S. aureus* that functioned with detectable efficiency when expressed in that background. The activity of phage- or host-encoded recombinase genes is considered to be highly dependent on the overall biology of an organism, including the presence of other contributory host factors and exonuclease systems (25). As such, it is generally thought that recombinases function with the highest efficiency in their background of origin (50). Our failure to identify a *S. aureus* recombinase which conforms to these expectations suggests either that recombinases native to *S. aureus* lack activity for ssDNA or that *S. aureus* recombinases which are optimal for recombineering are markedly dissimilar in sequence and structure to those which have been previously defined (51). We also found that certain *S. aureus* strains are naturally recombinogenic with mutagenic oligonucleotides introduced through electroporation. Although spontaneous, low-frequency ssDNA recombineering has been previously observed in *E. coli* (52), the substantial differences in spontaneous recombineering efficiency among *S. aureus* strains (Fig. 1C) suggest contributions of differing host genetic factors or physiologies in mediating this process.

Recombineering with CRISPR/Cas9 counterselection provides a novel tool for testing the phenotypic consequences of large- and small-scale genetic variation in the medically important organism *S. aureus*. Although robust in its current incarnation, future improvements to this technology may further improve or expand functionality. Definition and incorporation of factors which potentiate recombineering function in particular strain backgrounds could lead to further improved efficiencies of recombineering. Use of chemically modified oligonucleotides could enable bypass of the MMR system (53) without the need for accessory mutations or a *mutS*-deficient background (Fig. 4), the latter of which results in a mutator phenotype (47). Relatedly, expression of dominant-negative MMR proteins from the genome editing vectors could enable enjoyment of these benefits without necessitating disruption of chromosomal DNA repair genes (54). Development of a counterselection vector containing high-specificity mutants of Cas9 (55) could improve the specificity of counterselection for the related motifs which appear multiply in the *S. aureus* genome, whereas use of CRISPR effector protein Cpf1, which recognizes a 5′-TTTV-3′ PAM (56, 57), could expand counterselection to A/T-rich regions. Lastly, integration of functional elements from the recombineering and counterselection plasmids into a single element could further streamline the workflow, although the reduced transformation efficiency of that significantly larger plasmid could prove limiting (58).

In summary, we have developed a rapid and precise technology for genome editing in *S. aureus* which incorporates the use of recombineering, reported here for the first time in that organism. Leveraging recent advancements which enable exogenous DNA to be efficiently introduced into *S. aureus* strains (15, 16), this approach is broadly applicable across lineages, including poorly defined clinical isolates. In contrast to existing approaches, our technique is unique in utilizing commercially synthesized synthetic DNA oligonucleotides as substrates for introducing precise genomic modifications and performing counterselection, making it possible to scalably and inexpensively engineer desired changes. This method will facilitate studies seeking to address a variety of issues about the function of particular genes and specific mutations in *S. aureus*.

## MATERIALS AND METHODS

### Strains, plasmids, and whole-genome analysis.

Strains used in this study are summarized in Table S1 in the supplemental material. *S. aureus* strain ATCC 29213 (37) was obtained from the American Type Culture Collection and *S. aureus* N315 (40) from the Biodefense and Emerging Infections Research Resources Repository (BEI Resources). *S. aureus* strain N315ex w/o ϕ, from which the ϕ phage has been deleted (43), was a generous gift of Kazuya Morikawa (University of Tsukuba). Clinical *S. aureus* strains, each originating from a different patient, were obtained from the University of Washington Clinical Microbiology Laboratory as deidentified specimens. *Escherichia coli* SA08B (16) was obtained from Lucigen. *E. coli* DC10B (15) and *S. aureus* strain Newman (39) were generous gifts from Daniel Wolter (University of Washington).

Vectors pCN50 and pCN33 (36) were obtained from BEI Resources, and pCAS9 (27) and pCL52.2 (59) were purchased from Addgene.

All strains were maintained using LB media unless noted otherwise. *E. coli* carrying ampicillin resistance-encoding shuttle vectors was cultured at 37°C in the presence of 100 μg/ml antibiotic, *S. aureus* carrying chloramphenicol resistance vectors at 32°C in the presence of 10 μg/ml antibiotic, and *S. aureus* carrying erythromycin resistance vectors at 32°C in the presence of 10 μg/ml antibiotic. *S. aureus* strains carrying multiple vectors were maintained using both antibiotics. Rifampin resistance was selected using 25 μg/ml antibiotic.

Clinical strains were subjected to whole-genome sequencing and assembly as described elsewhere (60) and were assigned to sequence types using MLST v1.8 (61) and subsequently to clonal complexes using eBURST v3 (62).

### Vector construction.

Vectors generated in this study are summarized in Table S1. All oligonucleotides and synthetic gene sequences (Gblocks) were synthesized by IDT (Table S2). All restriction enzymes were obtained from New England Biolabs.

10.1128/mBio.00067-18.3 TABLE S2  Summary of oligonucleotides used. Download TABLE S2, XLSX file, 0.03 MB. Copyright © 2018 Penewit et al. 2018 Penewit et al. This content is distributed under the terms of the Creative Commons Attribution 4.0 International license.

The BsaI restriction site of pCN50 was first eliminated using site-directed mutagenesis with primers pCN_AMP_mut_F and pCN_AMP_mut_R to produce vector pCN50-BSAI.

Recombinases were synthesized as gBlocks under the control of the constitutive P23 promoter (63), and were codon optimized for *S. aureus*. gBlocks were digested with XmaI and NarI and cloned into similarly digested pCN50-BSAI to yield vectors pCN50-EF2132, pCN50-gp20, pCN50-recTS2, pCN50-recTS3, and pCN50-bet. Orientation of the recombinase as concordant with the pCN50 transcriptional terminator was confirmed by restriction digestion.

To generate vector pCN-EF2132tet, a *S. aureus* codon-optimized *tetR* cassette (64) under the control of the P23 promoter was synthesized as a gBlock and then amplified with primers tetR-Gibson_pcn50F and tetR-Gibson_pcn50R and Gibson assembled (65) into XhoI-digested pCN50-EF2132. Due to our inability to fully repress Cas9 expression under the influence of the *tetR* element, however, this feature was not relevant to the final implementation of our methods.

To construct vector pCAS9counter, the chloramphenicol resistance cassette of pCN50-BSAI was replaced with an erythromycin resistance cassette by amplifying *ermC* from vector pCN33 using primers 675_ermC_F and 675_ermC_R and cloning into ApaI and XhoI pCN50-BSAI restriction sites to generate plasmid pCN50-BSAI-emrc. The existing *S. aureus* replicon of pCN50-BSAI-ermc was replaced with the temperature-sensitive E194ts replicon of pCL52.2 by amplifying pCL52.2 with pE194ts_replicon_gibson_F and pE194ts_replicon_gibson_R, amplifying pCN50-BSAI-ermc with pcn50-ermc_repliconoverlap_F and pcn50-ermc_ repliconoverlap_R, and Gibson assembling the products to yield vector pCN50-ermc-E194ts. A minimal SsrA tag (34, 66) was introduced at the terminus of Cas9 in pCAS9 using site-directed mutagenesis with primers Cas9_ssRA_SDM_F and Cas9_ssRA_SDM_R, and then Cas9 was amplified from vector pCAS9 and placed under the control of the PRAB17 (49) promoter using primers cas9toprab17 and CAS9_r_V3, followed by cloning into pCN50-ermc-E194ts digested with XmaI and SbfI to yield pCN50-ermc-Cas9. Synthetic sgRNA (45) under the control of the PRAB17 promoter with a downstream transcriptional terminator was synthesized as a gBlock and cloned into the NarI site of pCN50-ermc-Cas9 to produce vector pCAS9counter.

pCAS9counter guide RNAs were designed using the program CRISPRscan (67). Guide RNAs were synthesized as ssDNA oligonucleotides (tailed 5′-AGCTC-3′ upstream and 5′-G-3′ downstream) and their reverse complements (tailed 5′-AAAAC-3′ upstream and 5′-G-3′ downstream), with tails allowing their ligation into the vector’s BsaI cut site. Oligonucleotides were phosphorylated and annealed in a reaction mixture containing using T4 PNK (New England Biolabs) in T4 ligase buffer heated to 37°C for 40 min and then to 95°C for 5 min and then gradually cooled to 20°C over 42 min. Annealed oligonucleotides were ligated into BsaI-digested pCAS9counter. The presence of targeting oligonucleotides was confirmed by fragment size shift (loss of ~100 bp) after amplifying with primers sgRNA_check_F and sgRNA_check_R.

### *S. aureus* transformation, recombineering, and counterselection.

Plasmids were made restriction compatible with *S. aureus* prior to electroporation by passaging in *E. coli* SA08B or *E. coli* DC10B. Electrocompetent cells were prepared essentially as described elsewhere (15), except that strains were cultured in either LB or B2 medium as necessary to support their growth.

For transformation of strains with recombineering vectors, electrocompetent cells were combined with 1 μg plasmid precipitated with pellet paint (Novagen) as described previously (15). Electroporation was performed using 1-mm-gap cuvettes and a Bio-Rad MicroPulser set to 2.3 kV and a 2.5-ms time constant. Following electroporation, strains were incubated in 950 μl recovery medium (15) at 32°C for 2 h before plating onto appropriate media was performed.

For recombineering experiments, mutagenic oligonucleotide was introduced into electrocompetent cells previously transformed with the recombineering vector. Mutagenic oligonucleotide (200 pmol in a 2-µl total volume) was combined with competent cells, and the cells were electroporated and grown as described above.

When recombineering was performed concurrently with counterselection, 200 pmol mutagenic oligonucleotide was used to resuspend 1 μg precipitated plasmid (15) in a 2-μl total volume, which was electroporated as described above into electrocompetent cells previously transformed with the recombineering vector.

### Data availability.

Genome assemblies from the six clinical isolates examined in this study have been deposited in NCBI GenBank under accession numbers PNPA00000000 through PNPF00000000.
