# Erratum for Penewit et al., “Efficient and Scalable Precision Genome Editing in Staphylococcus aureus through Conditional Recombineering and CRISPR/Cas9-Mediated Counterselection”

**DOI:** 10.1128/mBio.01839-18

**Published:** 2018-10-02

**Authors:** Kelsi Penewit, Elizabeth A. Holmes, Kathryn McLean, Mingxin Ren, Adam Waalkes, Stephen J. Salipante

**Affiliations:** a Department of Laboratory Medicine, University of Washington, Seattle, Washington, USA; b Department of Bioengineering, University of Washington, Seattle, Washington, USA

## ERRATUM

Vol. 9, issue 1, 10.1128/mBio.00067-18, 2018, https://doi.org/10.1128/mBio.00067-18. The first name of the third author was misspelled. The name should appear as shown above.

