# Erratum for Penewit et al., “Efficient and Scalable Precision Genome Editing in Staphylococcus aureus through Conditional Recombineering and CRISPR/Cas9-Mediated Counterselection”

**DOI:** 10.1128/mBio.02698-18

**Published:** 2019-01-15

**Authors:** Kelsi Penewit, Elizabeth A. Holmes, Kathryn McLean, Mingxin Ren, Adam Waalkes, Stephen J. Salipante

**Affiliations:** a Department of Laboratory Medicine, University of Washington, Seattle, Washington, USA; b Department of Bioengineering, University of Washington, Seattle, Washington, USA

## ERRATUM

Volume 9, no. 1, e00067-18, 2018, https://doi.org/10.1128/mBio.00067-18. We have discovered an error in Table S2 in the supplemental material. There was an incorrect name and sequence of the recombineering oligonucleotide listed in line 52. The sequence of the correct oligonucleotide (RpoBH481Y_NoPAM_V2) is now provided in the corrected table, which has been replaced online. We apologize for the inconvenience.

