# Supplementary material for: Efficient and Scalable Precision Genome Editing in Staphylococcus aureus through Conditional Recombineering and CRISPR/Cas9-Mediated Counterselection
Source: mBio. 2018 Feb 20;9(1):e00067-18. doi: 10.1128/mBio.00067-18 (PMC5821094; doi:10.1128/mBio.00067-18)
Supplement: FIG S1 [file mbo001183736sf1.pdf]

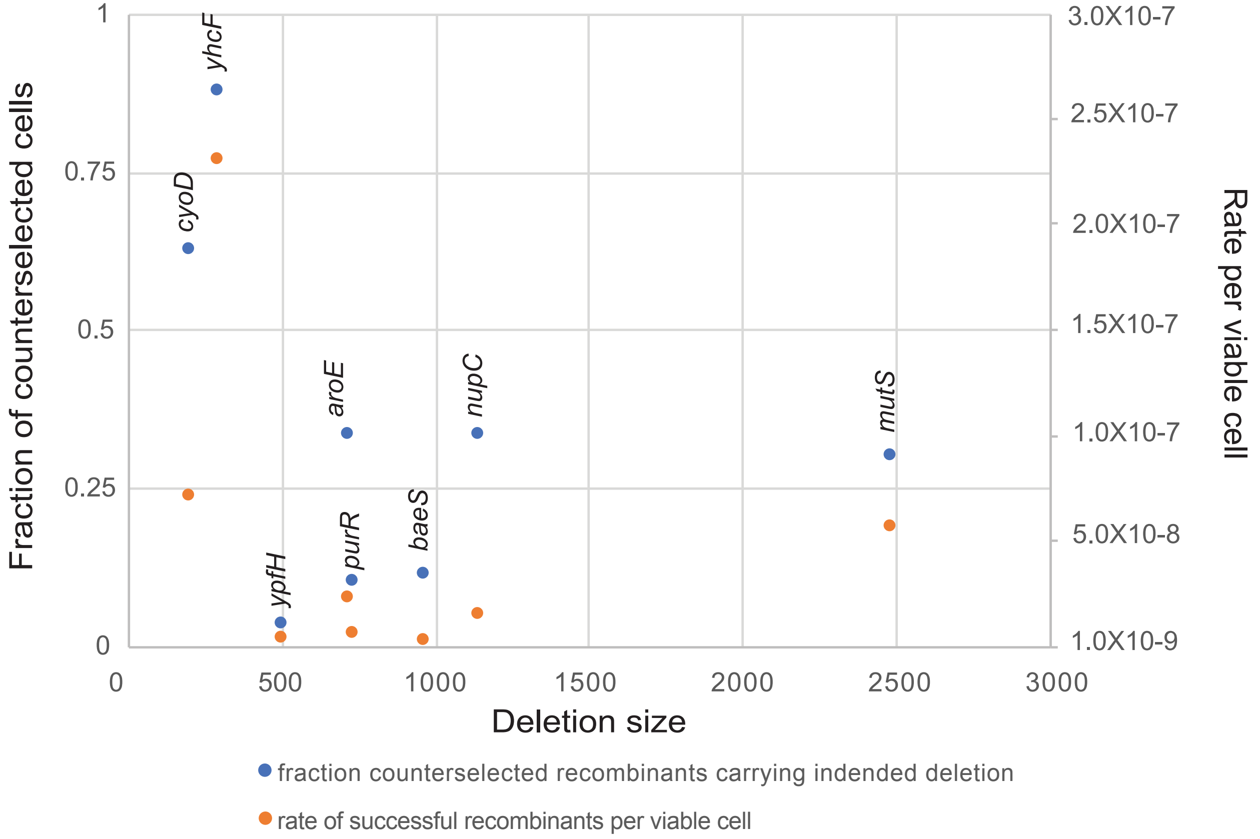

Fraction of counterselected cells
1
0.75
0.5
0.25
0
3.0X10-7
2.5X10-7
2.0X10-7
1.5X10-7
1.0X10-7
5.0X10-8
1.0X10-9
Rate per viable cell
yhcF
cyoD
aroE
nupC
mutS
ypfH
purR
baeS
0
500
1000
1500
2000
2500
3000
Deletion size
fraction counterselected recombinants carrying indended deletion
rate of successful recombinants per viable cell
